# Using the task-technology fit model to examine the use of telemedicine applications by general practitioners in Indonesia: A qualitative study

**DOI:** 10.1371/journal.pone.0351130

**Published:** 2026-06-08

**Authors:** Ni Luh Saddhwi Saraswati Adnyani, Rajesri Govindaraju, Khoirul Muslim, Titah Yudhistira

**Affiliations:** Faculty of Industrial Technology, Bandung Institute of Technology, Bandung, West Java, Indonesia; Touro University California College of Pharmacy, UNITED STATES OF AMERICA

## Abstract

Telemedicine has been recognized for improving healthcare accessibility and cost efficiency. However, telemedicine applications still face several limitations that must be addressed to better support users and their tasks. This study adopted the task-technology fit (TTF) model, encompassing task, technology, TTF, utilization, and performance impact aspects, to qualitatively explore general practitioners’ (GPs) perspectives and experiences regarding the use of telemedicine applications for remote healthcare services. Unlike prior telemedicine and TTF studies that predominantly employ quantitative approaches, this study provides an integrated qualitative examination of physicians’ clinical tasks, corresponding technology requirements, perceived TTF, utilization patterns, and performance impact of telemedicine applications, offering richer insights into real-world telemedicine use from physicians’ perspectives. Semi-structured interviews were conducted with 17 GPs experienced in remote consultations. Data were analyzed using content analysis. The findings show that GPs engage in a wide range of clinical tasks during telemedicine consultations. From a technological perspective, telemedicine applications must meet specific requirements to effectively support each of these tasks. Several critical application requirements were identified, particularly those related to clinical communication, data and service integration, and clinical task support. While telemedicine applications generally support physicians’ clinical tasks and contribute to improved efficiency, effectiveness, service quality, and physician–patient communication, significant gaps remain. This study offers valuable insights for telemedicine providers in designing strategies to optimize telemedicine applications in supporting physicians’ tasks, which in turn may enhance physicians’ utilization of telemedicine and improve their performance. In addition, policymakers and regulators may use the results to establish regulatory guidelines regarding the scope of services and standards for safe and standardized telemedicine practice.

## Introduction

The global healthcare system is rapidly transitioning from conventional models to inclusive digital solutions, driven by the Coronavirus Disease 2019 (COVID-19) pandemic and technological advancements [1,2]. This shift has led to the widespread adoption of telemedicine in addressing the increasing demands for healthcare [1]. In Indonesia, the implementation of telemedicine has grown significantly due to limited accessibility to healthcare services, particularly in rural and remote areas [1]. People living in rural and remote areas often experience difficulties in accessing healthcare facilities [3]. The shortage of medical professionals further compounds the issue. According to the Indonesian Ministry of Health, the physician-to-population ratio was only 0.62 per 1,000 people, which is below the World Health Organization (WHO) standard of 1:1,000 [4]. Moreover, most physicians are concentrated in Java, the most densely populated island, where the capital, Jakarta, is located [5], or in other urban areas, contributing to disparities in healthcare availability across the country [6]. In this context, telemedicine is expected to help bridge the gap by extending the reach of healthcare professionals and services to rural and remote areas that lack sufficient medical expertise and resources [3].

Telemedicine refers to the delivery of healthcare services at a distance using telecommunication technologies [7]. The benefits of telemedicine include improving the quality and accessibility of healthcare services, enhancing patient and physician satisfaction, decreasing unnecessary waiting times in hospitals, and reducing overall medical costs [8,9]. Telemedicine services are accessible through a digital application that allows patients to schedule appointments and consult virtually with physicians, purchase and receive medications, and access other healthcare services with ease [10].

To ensure the effective implementation and utilization of telemedicine applications, it is important to identify the current limitations or challenges of existing systems and find approaches to address them [11]. Common limitations found in telemedicine applications include poor audiovisual quality, which can hinder communication between patients and healthcare providers; difficulty in conducting physical examinations remotely, as certain essential elements, such as monitoring vital signs (e.g., measuring blood pressure), are challenging to perform virtually; and a lack of structured clinical data management [12]. A comprehensive evaluation of telemedicine applications is necessary to identify areas that need improvement or enhancement. The results of such evaluations can serve as a foundation for developing strategies to improve and optimize telemedicine applications.

Evaluating telemedicine from the perspective of physicians is essential, as they are a key stakeholder group in the implementation of telemedicine services. Although telemedicine involves various stakeholders [13], including government, entrepreneurs, patients, and general hospital staff, physicians represent the largest stakeholder group in the delivery of healthcare services [14]. Assessing telemedicine applications from the physician’s perspective is not only essential to ensure that these applications meet the necessary standards for remote healthcare services to be comparable to in-person care, but also to ensure that their use enhances the quality, efficiency, and effectiveness of healthcare delivery.

One framework that can be used to evaluate telemedicine applications is the task-technology fit (TTF) model, which assesses whether telemedicine applications effectively meet user needs and support their tasks. The TTF model is a simplified form of the Technology-to-Performance Chain (TPC) model developed by Goodhue and Thompson [15], focusing primarily on the relationship between task and technology and its influence on utilization and performance [16].

TTF represents the degree to which a technology supports individuals in performing their tasks [15,17]. Previous studies have examined TTF in the context of telemedicine from various perspectives, such as physicians [18–20], nurses [21], and patients [10,22–24]. Most of these studies primarily focused on technology adoption or usage continuance [10,19–24], while the performance impact of technology use, especially from the perspective of the physician, has received limited attention. The TTF model, however, provides a more holistic lens for understanding the impact of technology on individual-level performance [15]. The premise of the TTF model is that technology must not only be used but also must fit the task or enable the user to perform the task in order to have a positive performance impact [15,25,26].

In addition, most of the existing studies that examine TTF in the context of telemedicine primarily employed quantitative methods [10,19–23], which are valuable for testing the existence and strength of relationships among variables and even for making predictions based on those relationships [27]. Nonetheless, such approaches fall short in capturing the richness of user experiences and the contextual factors that influence technology use. In contrast, qualitative approaches allow for an in-depth exploration of individual experiences, preserving the high level of detail and nuance in each participant’s perspective [27].

Accordingly, there remains a clear research gap in the telemedicine and TTF literature. Prior studies have rarely examined, in an integrated manner, physicians’ clinical tasks, the corresponding telemedicine application requirements, perceived TTF, utilization patterns, and performance impact of telemedicine applications using an in-depth qualitative approach. To address this gap, this study applies the TTF model to explore physicians’ perspectives and experiences regarding the use of telemedicine applications for delivering remote healthcare services (clinical consultations) to patients. The aim is to expand upon previous findings by providing a current and theory-based perspective on the use of telemedicine applications from the viewpoint of physicians in Indonesia.

To the best of our knowledge, this is the first qualitative study in Indonesia, a developing country [28], to comprehensively examine the task, technology, TTF, utilization, and performance impact aspects of telemedicine applications from the physician’s perspective. A qualitative approach was employed, as it allows for an in-depth and detailed exploration of relevant issues [29]. This deeper insight is valuable for revealing how telemedicine applications function in real-world clinical settings, uncovering specific issues or unmet needs that may not be captured through quantitative metrics alone. By capturing physicians’ experiences, qualitative findings can guide targeted improvements to better support clinical workflows and ultimately enhance the quality of remote healthcare service delivery.

General practitioners (GPs) were chosen as the focus of this study. GPs are medical doctors who provide primary, continuous, and comprehensive whole-person care to individuals, families, and the community [30]. They work in the front line of the healthcare system and take the initial steps in providing care for any health problems patients may have [31,32]. As primary care providers, GPs encounter a wide range of health issues, giving them extensive experience in patient interaction.

Therefore, the primary objective of this study is to examine the use of telemedicine applications by GPs in delivering remote healthcare services through the lens of the TTF model. Specifically, this study aims to explore (1) the clinical tasks performed by GPs in telemedicine-based consultations, (2) the application requirements needed to support these tasks, (3) GPs’ perceptions of the TTF of telemedicine applications, (4) telemedicine application utilization, and (5) the performance impact of telemedicine application use.

## Materials and methods

### Theoretical framework

The TTF model developed by Goodhue and Thompson [15] explains how technology can lead to performance impact at the individual level. The key components of this model include task, technology, TTF, utilization, and performance impact. Goodhue and Thompson [15] define TTF as the extent to which a technology facilitates individuals to perform their tasks. In the context of telemedicine practice, TTF can be defined as the degree to which telemedicine applications support physicians in performing remote healthcare delivery tasks effectively [20]. TTF focuses on the utility of the technology for meeting the needs of the task at hand, the deficiency of which could lead to inefficiencies and compromised quality of care [33].

The antecedent of TTF is the interaction between task and technology [21]. Tasks are broadly defined as actions performed by individuals to transform inputs into outputs, while technology is viewed as a tool used by individuals to carry out their tasks [16]. In telemedicine settings, physicians’ tasks include patient assessment through anamnesis, physical examination via audiovisual interaction, diagnosis establishment, medication prescribing, and follow-up consultations [20,34–37]. Goodhue and Thompson [15] state that specific types of tasks require specific technological functions. The greater the gap between task requirements and the functions provided by the technology, the lower the TTF. Organizations should prioritize task and technology alignment to optimize TTF, ensuring technologies meet task-specific needs [38]. Accordingly, the telemedicine application used by physicians in providing remote health services must be able to support the clinical tasks required to provide such services.

In the TTF model, Goodhue and Thompson [15] describe the relationship between TTF, technology utilization, and the impact of technology use on individual performance (performance impact). In general, utilization refers to the act of using a technology as a tool or resource to complete specific tasks [39]. Utilization is not limited to general technology use but includes several specific aspects such as routine use, feature use, and value-adding use [40]. In the context of this study, utilization refers to the extent to which telemedicine applications are used by physicians in their remote healthcare delivery tasks.

Performance impact refers to the extent to which the use of a particular technology enhances an individual’s effectiveness, efficiency, and productivity in performing their tasks [15]. In telemedicine practice, performance impact may include improvements in quality of patient care [40] and healthcare efficiency [41] from telemedicine application use.

The TTF model emphasizes that neither utilization nor TTF alone is sufficient to fully explain the performance impact of a technology [42]. According to Goodhue and Thompson [15], more frequent use of a system does not necessarily result in higher performance; using a poor system, one with a low TTF, will not improve performance. Conversely, a model that focuses solely on TTF does not adequately account for the fact that the system must first be used in order to have any impact on performance [15]. Accordingly, this study adopts the TTF model as an integrated framework. In this study, the variables from the TTF model—namely, task, technology, TTF, utilization, and performance impact—were explored and used as the foundation for developing an interview guide (see S1 File), ensuring that physicians’ telemedicine experiences were systematically explored in relation to each TTF component, thereby providing a coherent theoretical basis for the qualitative inquiry.

### Study design and setting

This study employed a qualitative design, allowing for an in-depth exploration of GPs’ experiences and views regarding the use of telemedicine applications in providing remote healthcare services. Data were collected through qualitative interviews using open-ended questions. One advantage of qualitative interviews is their interactive nature, which enables unanticipated topics to surface and be explored further by the researcher [43].

The setting of this study was the use of telemedicine applications by GPs to deliver remote healthcare services (clinical consultations) to patients. This study was conducted in collaboration with Good Doctor, a technology-based integrated healthcare provider that offers telemedicine services to deliver quality healthcare access to users across Indonesia. As one of the telemedicine service providers in Indonesia, Good Doctor has a network of physicians who are actively involved in delivering remote healthcare services to patients.

### Participant recruitment

Interview participants were selected using the purposive sampling method, in which participants were intentionally chosen based on predetermined criteria relevant to the research objectives [44]. One of the main criteria was that participants had to be GPs with experience using telemedicine applications to provide remote healthcare (clinical consultations) to patients.

In this study, the Vice President (VP) of Medical Operations at Good Doctor was responsible for the participant selection process by identifying and assigning GPs who met the predefined inclusion criteria to take part in the interviews. The role of the VP was limited to coordinating and scheduling interviews based on the availability of eligible participants and the principal investigator. The research team did not influence the selection of specific individuals beyond confirming eligibility, and participants were interviewed in the order of availability from the pool of eligible physicians. Because participants were assigned based on availability rather than specific personal or professional attributes, the risk of selection bias was considered minimal. This approach was adopted to ensure feasibility while maintaining neutrality in participant recruitment.

Interviews were conducted until data saturation was reached. Data saturation refers to the point in data collection when new insights are no longer identified and the data begin to repeat, making further data collection redundant and indicating that a sufficient sample size has been achieved [45]. According to Stamer et al. [46], this is the most commonly used approach in qualitative research to assess sample size adequacy.

Hennink and Kaiser [45] identified several strategies for assessing saturation, including code frequency counts, comparative method, stopping criterion, high-order groupings, and code meaning. In this study, data saturation was assessed using the code meaning approach. This approach does not rely on counting the frequency of codes but instead focuses on achieving a comprehensive understanding of each code. It involves reviewing an interview and noting each issue (or code) identified, then in subsequent interviews assessing whether any new aspects, dimensions, or nuances of that code are identified, until no additional insights were observed and the codes were considered saturated [45].

Based on this analytical assessment, saturation was achieved after 17 interviews. All interviews were conducted via video calls using the Microsoft Teams software.

### Data collection

Semi-structured interviews were conducted between 19 June 2024 and 11 September 2024. This approach ensured that core questions were consistently asked in each interview while allowing flexibility to add follow-up or in-depth questions when necessary.

Prior to conducting the interviews, an interview guide was developed containing a list of questions or topics to be explored during the interviews. The guide was created based on relevant literature and reviewed by experts before being used in the interviews with the participants. A pilot test of the interview guide was conducted with one general practitioner (GP). Based on the pilot test, no major modifications were made; however, the guide was refined iteratively throughout the study as needed. For example, if during an interview a GP mentioned an additional requirement that should be provided in a telemedicine application to support a specific task, a related question would be added to the interview guide for subsequent use.

The interviews were conducted by the principal investigator (NLSSA), a female lecturer and PhD student who is currently conducting research on the task-technology fit of telemedicine applications from the perspective of the physician. She had undergone training and studied the qualitative research methodology literature, including interview techniques and content analysis. Additionally, she consulted with experts experienced in qualitative research to ensure the use of appropriate approaches in data collection and analysis.

The principal investigator had no prior relationship with the participants. At the time of the interviews, the participants were informed that the principal investigator was a lecturer and PhD student, and that the interviews were part of her PhD research. The interviews were audio-recorded and lasted between 60 and 99 minutes. No other individuals were present during the interviews; only the participant and the principal investigator took part in each session to maintain privacy and ensure that participants felt comfortable sharing their experiences.

### Data analysis

The interview recordings with GPs were transcribed by the principal investigator. All transcripts were imported into Atlas.ti to facilitate data analysis. The transcripts were then analyzed using content analysis. Content analysis can be applied to qualitative and quantitative data and may be conducted using an inductive or deductive approach [47]; the choice of approach depends on the research objectives. In this study, similar to the approach used by Stamer et al. [46], initial categories were developed deductively based on the interview guide or protocol and were then supplemented with additional categories formed inductively from the data coded from the transcribed interviews.

Content analysis consists of three main phases: preparation, organizing, and reporting. The preparation phase begins with the selection of the unit of analysis [47]. In this study, the unit of analysis was the interview text related to the use of telemedicine applications by GPs. In addition to determining the unit of analysis, the preparation phase also involves deciding whether to analyze only the manifest content or also the latent content. This study focused on the manifest content, which emphasizes what is explicitly stated in the text [47], allowing for more objective interpretation. The next step in the preparation phase was to become thoroughly familiar with the data and understand what was happening to gain a comprehensive understanding. Therefore, the interview transcripts were read repeatedly [47].

**Fig 1:**
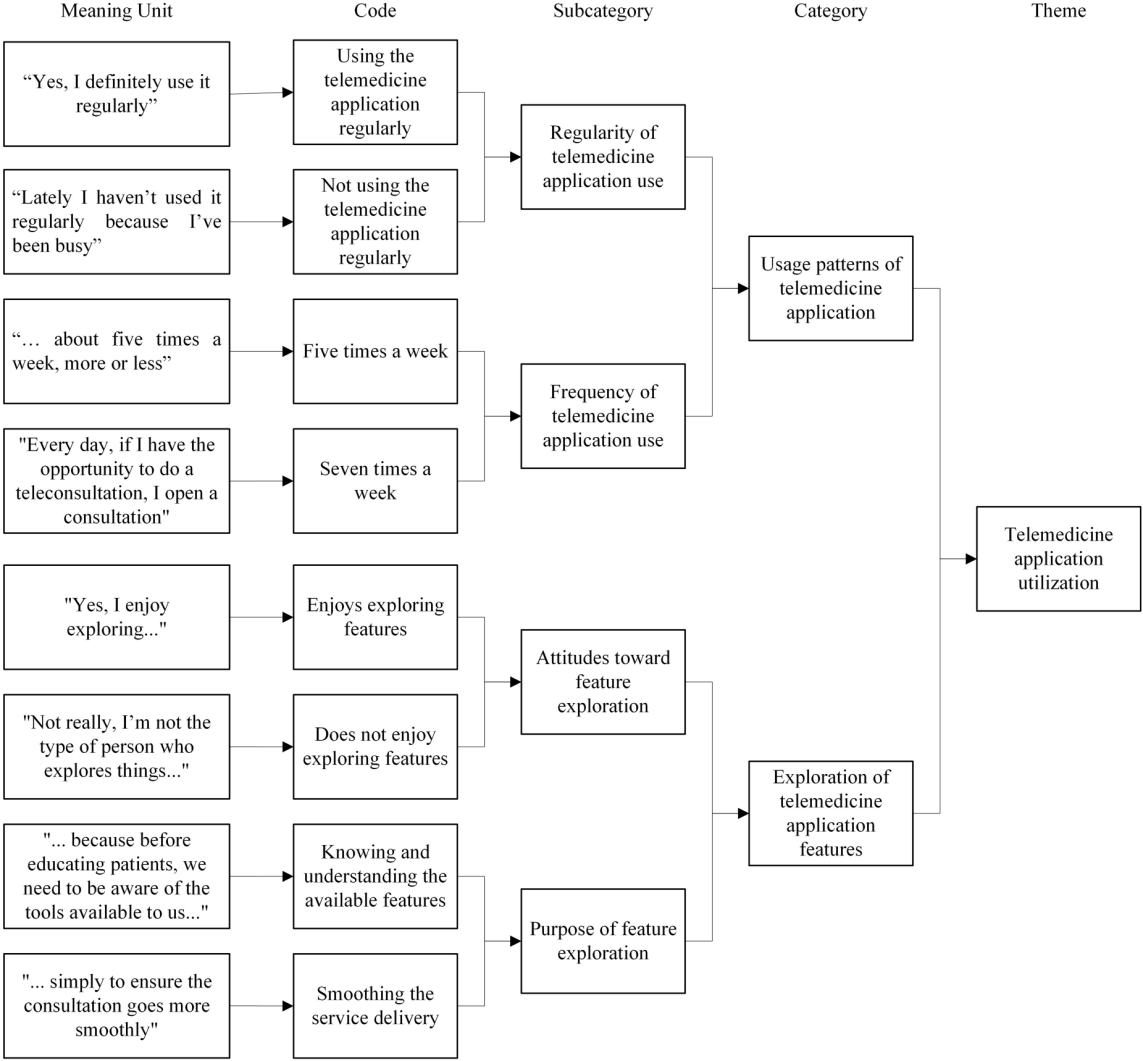
An example of the content analysis process illustrating the progression from meaning units to themes.

The next phase in content analysis is the organizing phase. In this phase, the interview text is divided into meaning units that address the same issue [48]. Each meaning unit is labeled with a code, a name that accurately describes the content of the corresponding meaning unit [49]. In deductive analysis, meaning units are coded based on a pre-developed categorization matrix, while in inductive analysis, they are coded openly through an open coding process. These codes are then grouped into subcategories based on their similarities or connections. The subcategories are further organized into categories and eventually into themes. An example of the progression from meaning units to the formation of themes in the content analysis process is given in Fig 1. Each code, subcategory, category, and theme was assigned a name and a brief description.

Theme validation was conducted through an iterative review process in which codes, subcategories, categories, and themes were repeatedly examined to ensure logical consistency and analytical coherence across all levels of analysis. This entire process was carried out by the principal investigator, who also regularly discussed the data analysis results with the research team to obtain feedback. As the data analysis was conducted by a single researcher, formal inter-coder reliability testing was not performed.

The final phase in content analysis is the reporting phase. In this phase, a detailed description of the data analysis process is provided, along with a systematic presentation of the findings [47].

### Ethical considerations

The Head of Ethics Subcommittee at Good Doctor (Decree No.: 001/KOMDIK/VI/2024) approved the study design and procedures. Before each interview began, the participant was provided with a brief explanation of the study, including the purpose and procedures of the interview, their right to withdraw at any time without any consequences, and the assurance of confidentiality regarding the information they provided. Once participants had given their verbal consent to be interviewed, the interview commenced. Verbal consent was obtained rather than written because the interviews were conducted remotely, making it impractical to obtain signed forms. All verbal consents were audio-recorded and served as documentation, with the interviewer present as the witness. No participants refused to participate or withdrew from the study. To protect participants’ privacy, each participant was assigned a unique code during the transcription process to ensure the confidentiality of their identity. The interview transcripts were accessible only to the research team.

### Rigor and trustworthiness

To ensure rigor, this study was reported following the Consolidated Criteria for Reporting Qualitative Research (COREQ) developed by Tong et al. [50] (see S1 Checklist). In addition, following Ylitörmänen et al. [48], the criteria of credibility, confirmability, dependability, and transferability proposed by Lincoln and Guba [51] were applied to ensure trustworthiness. The interviewer built trust by providing the participants with clear information about the study and the interview process. Credibility was maintained by giving the participants sufficient time to respond without interruption and by actively listening to their responses. However, the interview transcripts were not returned to the participants for review, nor did the participants provide feedback on the findings, as the interviewer interacted with them solely online via Microsoft Teams links provided by Good Doctor and therefore did not have direct access to the participants’ contact information. Nevertheless, the transcriptions were carefully completed based on the recorded interviews to ensure data accuracy. Confirmability was achieved through a transparent analytical process, including the presentation of examples to illustrate how meaning units were developed into subcategories, categories, and themes. Dependability was strengthened through discussions with the research team during the data analysis process. Additionally, dependability was supported by the use of qualitative data analysis software (Atlas.ti) to ensure systematic data analysis and track code frequency. Transferability was supported by providing detailed and accurate descriptions of the analysis process and the study findings.

## Results

The demographic characteristics of the physicians interviewed in this study are presented in Table 1. Interviews were conducted with 17 GPs. In terms of telemedicine application usage, 47.06% of participants had used telemedicine applications for 5–7 years, and another 47.06% for 2–4 years. Only 5.88% had less than 2 years of experience using telemedicine applications. All participants in this study had used the Good Doctor application (100%). In addition, some participants also had experience using other telemedicine platforms, such as Halodoc (29.41%), Alodokter (17.64%), YesDok (5.88%), Klik Dokter (5.88%), and other platforms (11.76%).

Based on the content analysis, several themes and categories were identified, as shown in Table 2. These themes correspond to the most relevant constructs of the TTF model. The following describes the themes and categories identified based on the content analysis.

**Table 1:** Demographic characteristics of interview participants.

Characteristic	Category	Frequency	Percentage
Type of Medical Practice	General Practitioner	17	100%
Gender	Male	9	52.94%
	Female	8	47.06%
Age	30-39 years	14	82.35%
	40-49 years	2	11.76%
	50-59 years	1	5.88%
Years of Practice as a Physician	1-9 years	10	58.82%
	10-18 years	5	35.29%
	19-27 years	2	5.88%
Years of Using Telemedicine Applications	2-4 years	8	47.06%
	5-7 years	8	47.06%
	<2 years	1	5.88%
Telemedicine Applications Previously and/or Currently Used	Good Doctor	17	100%
	Halodoc	5	29.41%
	Alodokter	3	17.64%
	YesDok	1	5.88%
	Klik Dokter	1	5.88%
	Others	2	11.76%

**Table 2:** Themes and categories.

Themes	Categories
Clinical Tasks Performed by GPs in Remote Healthcare Services Delivery	Anamnesis
	Conducting physical examinations
	Providing advice or education
	Establishing diagnoses
	Providing treatment plans
	Prescribing medications and/or medical devices
	Issuing referrals
	Conducting follow-ups^a^
	Reviewing diagnostic test results^a^
	Issuing medical certificates^a^
Telemedicine Application Requirements (Req.)	Req. for anamnesis
	Req. for conducting physical examinations
	Req. for providing advice or education
	Req. for establishing diagnoses
	Req. for providing treatment plans
	Req. for prescribing medications and/or medical devices
	Req. for issuing referrals
	Req. for conducting follow-ups^a^
	Req. for reviewing diagnostic test results^a^
	General requirements
Task-Technology Fit (TTF) of Telemedicine Application	TTF evaluation of telemedicine application
	Patient conditions that cannot be managed through telemedicine application
Telemedicine Application Utilization	Utilization of telemedicine application features
	Exploration of telemedicine application features
	Usage patterns of telemedicine application
Performance Impact of Telemedicine Application	Aspects of performance considered important
	Performance that can/cannot yet be improved
	Suggestions to improve GPs’ performance

^a^ Inductively derived category.

### Theme one: Clinical tasks performed by GPs in remote healthcare services delivery

Based on the Decree of the Minister of Health of the Republic of Indonesia Number HK.01.07/Menkes/4829/ 2021 regarding Guidelines for Health Services through Telemedicine during the Coronavirus Disease 2019 (COVID-19) Pandemic, several health services can be provided via telemedicine, one of which is clinical consultation, which includes the following tasks: anamnesis; conducting physical examinations through audiovisual means; providing advice or education needed based on the results of diagnostic test and/or physical examinations; establishing diagnoses; providing treatment plans; prescribing medications and/or medical devices; and issuing referral letters for further examinations or treatments to laboratories and/or other health facilities based on patient management outcomes. Interviews with GPs were conducted based on this list of tasks to determine which tasks are commonly performed by GPs in providing remote health services through telemedicine applications and to identify any additional tasks that may be needed.

Based on the interviews with GPs, all GPs confirmed that anamnesis is always conducted in the provision of remote health services. Anamnesis involves gathering a patient’s medical history through questioning, which includes their personal and family history, current health issues, and lifestyle factors, to aid in diagnosis and treatment planning.

It is a fundamental task that is consistently performed, as expressed by one GP:

*“Anamnesis is definitely essential. It’s fundamental because from anamnesis we can discern the likely direction of the disease.”*

Physical examinations, which involve the evaluation of specific physical conditions in patients through audiovisual media, were conducted by all GPs. However, some GPs believe that physical examinations are not always necessary and are only performed when certain patient conditions necessitate a more detailed physical evaluation. One GP conveyed:

*“Physical examinations are sometimes necessary and sometimes not, only under specific conditions.”*

However, there are still challenges in conducting physical examinations through telemedicine applications. Several GPs reported that physical examinations are difficult to perform through audiovisual means and can only be conducted to a limited extent. Additionally, there are specific conditions where certain physical examinations are not feasible, as mentioned by one GP:

*“In traditional physical examinations, we typically perform procedures such as auscultation using a stethoscope, palpation by feeling with hands, and percussion by tapping on specific areas of the body to detect signs like meteorism or other abnormalities. However, these procedures are not feasible during telemedicine consultations.”*

Most GPs reported that physical examinations of patients are often facilitated using photographs or other visual aids. In cases requiring a physical examination, GPs typically ask patients to upload images or provide a detailed description of their condition. Additionally, some GPs noted that certain physical examinations can be conducted independently by patients, who then report the results back to the GP. The use of personal medical devices owned by patients, such as blood pressure monitors or glucometers, was considered particularly helpful in supplying additional relevant information, as explained by one GP:

*“... if we can assess the cases from photos, it simplifies the diagnostic process… Sometimes, if patients have their own blood pressure monitors, they can inform us of the readings, or if they have glucometers, they can provide that information as well. This means that physical examinations that can be performed remotely by patients themselves are quite helpful.”*

Providing advice or education, which involves offering clinical guidance or patient education based on the results of physical examinations and/or supporting diagnostic tests, is a key task performed by GPs in delivering remote healthcare services through telemedicine applications. All GPs confirmed that they provide advice or education to patients as part of their telemedicine services. As one GP stated:

*“The first thing that is always done is anamnesis, followed by the provision of advice.”*

Establishing a diagnosis, the process by which GPs determine the type of disease or health condition of a patient by analyzing symptoms based on information obtained from anamnesis, physical examination, and/or supporting diagnostic tests, is recognized by all GPs as a task performed in the delivery of remote health services via telemedicine applications. However, there are limitations to the types of diagnoses that can be made, as highlighted by one GP:

*“... the diagnosis we make might be provisional rather than definitive… So, in clinical settings, there are provisional and definitive diagnoses. Establishing a definitive diagnosis requires results from supporting diagnostic tests such as X-rays or ultrasounds. Actually, it is the same as what we do offline in primary care facilities.”*

Providing treatment plans involves clinical decisions made by GPs based on diagnoses, which may include recommendations for pharmacological and non-pharmacological therapies, as well as referrals or further medical procedures tailored to each patient’s specific condition. The interviewed GPs confirmed that they provide treatment plans to patients as part of delivering remote healthcare services via telemedicine applications. This also includes prescribing the necessary medications based on diagnoses. One GP mentioned:

*“... we are also involved in providing treatment plans and medications by prescribing through the app. The prescription is entered into the system, and we handle that as well.”*

However, prescribing medical devices is rarely done. One GP stated:

*“We rarely prescribe medical devices, as they are not included in the system.”*

Referral refers to the act of directing patients to laboratories or other healthcare facilities for further examination or treatment. Referral is one of the tasks performed by GPs in telemedicine services. However, some GPs noted that referral letters are issued only when deemed necessary, depending on patient conditions, as explained by one GP:

*“… the issuance of referrals is heavily dependent on the diagnosis, so if there are any warning signs, we are obliged to refer patients to specialists or for further examinations at the lab.”*

As part of the discussion on tasks commonly performed in delivering remote healthcare services via telemedicine applications, in addition to reviewing the tasks listed in the Decree of the Minister of Health of the Republic of Indonesia Number HK.01.07/Menkes/4829/2021, the participants were also asked to suggest any additional tasks they considered important. These suggestions were then confirmed with other GPs during subsequent interviews. The additional tasks proposed by the participants included conducting follow-up consultations and reviewing diagnostic test results.

Follow-up is the process of ongoing monitoring and care of patients after their initial treatment, carried out by GPs to evaluate the effectiveness of treatment, detect side effects, and ensure the patient’s recovery progress. Based on interviews, several participants stated that they conduct follow-ups as part of remote healthcare services via telemedicine applications and emphasized that follow-up is an essential task for physicians. However, some participants believed that follow-ups are only required for certain cases. One GP expressed:

*“Follow-ups are actually necessary. However, the need for follow-ups really depends on the patient’s condition. For example, if we prescribe medication for high blood pressure, a follow-up is indeed required, or if we prescribe diabetes medication, we need a follow-up to determine whether the medication is effective or causing any side effects.”*

Some participants confirmed that they review the results of diagnostic tests as part of remote healthcare services provided via telemedicine applications. This review serves as an additional assessment to confirm or strengthen a diagnosis initially established through anamnesis and physical examination. Nevertheless, some participants stated that this task is only necessary in specific cases, and the type of diagnostic tests that can be reviewed is limited to laboratory test results. To have more complex diagnostic tests (e.g., X-rays, ultrasounds, CT scans, or MRIs) reviewed, patients typically need to be referred to specialists, as GPs generally do not have the expertise to review or interpret such tests. One GP said:

*“If a review of diagnostic tests is needed, we can only assist with interpreting and providing treatment based on lab results, because if it involves X-rays or other complex diagnostic tests like ultrasound, CT scan, or MRI, a referral to a specialist for further management is required.”*

Based on the interview results, several participants mentioned that they issue medical certificates, commonly serving as sick notes, as part of the remote healthcare services provided through telemedicine applications. Issuing medical certificates involves providing an official document stating that an individual requires rest and is temporarily unable to work or carry out regular activities due to health-related reasons. As one GP stated:

*“… even now, if necessary, we can issue a medical certificate to patients from our app.”*

### Theme two: Telemedicine application requirements

To facilitate the collection of information from GPs regarding the requirements needed in a telemedicine application to support their clinical tasks, a preliminary identification of these requirements was conducted prior to the interviews based on literature studies. During the interviews, the participants were asked to confirm whether the identified requirements were required and were also given the opportunity to suggest any additional requirements they considered important. These suggestions were subsequently validated with other GPs in later interviews. Furthermore, if the participants proposed any additional tasks during the interview, they were also asked to specify the requirements needed in the telemedicine application to support those tasks. A summary of the final results from the requirement identification process is presented in Table 3, while the description of each identified telemedicine application requirement is provided in S1 Table.

**Table 3:** Telemedicine application requirements.

Task	No.	Requirement	Required^a^	Requirement Groups
Anamnesis	1	Real-time communication	17/17	Clinical communication
	2	Upload images	16/17	
	3	Upload documents	14/17	
	4	Access to patient data	16/17	Data and services integration
	5	Integration with other platforms	12/17	
	6	Patient medical data recording	17/17	
	7	Question template^b^	2/2	Clinical task support
Conducting physical examinations	1	Record and store	17/17	Data and services integration
	2	Integration with medical devices	16/17	
	3	Real-time communication	15/17	Clinical communication
	4	Upload short audio^b^	4/5	
	5	Upload images^b^	9/11	
	6	Upload short video^b^	10/12	
Providing advice or education	1	Integration of examination data	16/17	Data and services integration
	2	Referral function	17/17	
	3	Access to healthcare facility data	16/17	
	4	Advice/education documentation	17/17	
	5	Recommendation function	16/17	Clinical decision support
	6	Real-time communication	17/17	Clinical communication
Establishing diagnoses	1	Recommendation function	15/17	Clinical decision support
	2	Real-time communication	17/17	Clinical communication
	3	Collaboration function	9/17	
	4	Medical data access and integration	17/17	Data and services integration
	5	Access to medical database	17/17	Clinical task support
	6	Reporting feature	17/17	
	7	Complete ICD-10 list^b^	5/7	
Providing treatment plans	1	Prescription recording	17/17	Clinical task support
	2	Patient monitoring	16/17	
	3	Referral function	17/17	
	4	Treatment notes	17/17	
	5	Recommendation function	15/17	Clinical decision support
	6	Real-time communication	17/17	Clinical communication
	7	Collaboration function	11/17	
	8	Feedback and evaluation	15/17	
	9	Informed consent^b^	5/9	
Prescribing medications and/or medical devices	1	Prescription template	17/17	Clinical task support
	2	Open loop prescribing	9/11	
	3	Electronic prescription identification code	13/17	
	4	One-time use	17/17	
	5	Access to medications and/or medical devices data	16/17	Data and services integration
	6	Electronic prescription documentation	17/17	
	7	Closed-loop prescribing	9/11	
	8	Integration with pharmacy facilities	17/17	
	9	Recommendation function	17/17	Clinical decision support
	10	Automated medication restriction for drugs excluded by Ministry of Health regulations	17/17	
	11	Automated medication restriction to prevent the prescription of drugs that are contraindicated for specific patient conditions^b^	11/11	
Providing referrals	1	Referral letter template	17/17	Clinical task support
	2	Access to patient data	17/17	Data and services integration
	3	Integration with healthcare facilities	17/17	
	4	Referral history	17/17	
	5	Collaboration function	15/17	Clinical communication
Conducting follow-ups	1	Follow-up note^b^	5/6	Clinical communication
	2	Real-time communication^b^	5/7	
	3	Upload images^b^	5/6	
	4	Reminder^b^	7/8	Scheduling and notification
	5	Follow-up scheduling^b^	2/2	
Reviewing diagnostic test results	1	Referral function^b^	5/5	Data and services integration
	2	Diagnostic test history^b^	3/3	
	3	Upload documents^b^	6/6	Clinical communication
	4	Upload images^b^	7/7	
General task	1	System reliability	17/17	System reliability
	2	Ease of use	17/17	Ease of use and support
	3	Assistance	17/17	
	4	Authorization	17/17	Privacy and security
	5	Privacy and security	17/17	
	6	Right level of detail	17/17	Data quality and accessibility
	7	Currency	17/17	
	8	Accuracy	17/17	
	9	Compatibility	17/17	
	10	Accessibility	17/17	
	11	Locatability	17/17	
	12	Presentation	17/17	
	13	Notification	17/17	Scheduling and notification
	14	Scheduling	16/17	

a Values are shown in the format x/y. The numerator (x) indicates the number of physicians who stated that the requirement is necessary, while the denominator (y) indicates the total number of physicians who were asked about that specific requirement. For example, 15/17 means that 15 out of 17 physicians who were asked about this requirement agreed that it is necessary.

b The requirement was added by the participant.

Based on the interview findings, several requirements were found to support multiple tasks. Therefore, the identified requirements were classified into several groups, namely data and services integration, clinical decision support, clinical communication, clinical task support, scheduling and notification, system reliability, ease of use and support, privacy and security, along with data quality and accessibility. A detailed classification of these requirements into groups is presented in Table 3.

As shown in Table 3, several telemedicine application requirement groups emerged as particularly critical across multiple clinical tasks. First, clinical communication was consistently emphasized as essential for almost all tasks. For example, in the task of providing treatment plans, one GP highlighted the importance of clear communication when explaining prescribed medications to patients:

*“When we prescribe, we should explain to the patient how to use the medication, for how many days, and whether certain drugs should be taken long-term or finished. For some critical medications, we also need to inform patients about key warnings, for example, that cold medicine may cause drowsiness.”*

Second, data and services integration, including access to medical records and integration with external platforms such as laboratories and pharmacies, was identified as fundamental for ensuring continuity of care. One GP emphasized the importance of integrated patient medical records:

*“Access to patient medical records is necessary; it is already in place and mandatory, especially when patients have a history of allergies or other medical conditions.”*

Third, clinical task support, such as structured prescription templates and referral letter templates, was viewed as critical for supporting efficient clinical workflows, particularly medication prescribing and referrals. One GP noted:

*“Prescription templates are needed, and they are already available here.”*

The task of issuing medical certificates emerged as one of the tasks identified during interviews with GPs. However, unlike other tasks, it was not explicitly proposed by the interviewees when they were asked to identify additional tasks necessary for providing remote healthcare services to patients. Instead, it was indirectly recognized through discussion with the participants as they responded to other interview questions. Since this task was not deliberately raised by the GPs, they were not specifically asked about the system requirements needed to support it. Consequently, no specific requirements related to the issuance of medical certificates were identified from the GPs’ perspective in this study.

### Theme three: Task-technology fit of telemedicine application

Based on the interviews with GPs, most stated that telemedicine applications adequately support their tasks in delivering remote healthcare services. One GP explained:

*“Yes, it can help in providing remote healthcare services because the application already includes everything needed. For example, we can chat with patients and they can send pictures, which also aids in diagnosis. Through the application, we can now provide both medication and non-medication recommendations, as well as advice on appropriate actions to take. If necessary, we can now issue referrals to labs or specialists and if needed we can also issue a sick note for patients through the application.”*

However, one GP revealed that although telemedicine applications support the tasks involved in providing remote healthcare services, there are still shortcomings that need to be addressed. The GP mentioned:

*“Yes, well, it’s sufficient, but there are still shortcomings, such as the inability to provide integrated referrals for lab tests, other diagnostic tests, and referrals to specialists or the nearest hospital—there are still shortcomings there.”*

Moreover, some participants explained that certain patient cases cannot be appropriately managed through telemedicine services. Telemedicine services are not suitable for patients who require immediate treatment at a healthcare facility or have complaints that necessitate thorough physical examinations or diagnostic tests, as one GP said:

*“Patients with emergency conditions, such as falls or accidents, cannot be examined via telemedicine because they require direct examination. Similarly, chest pain that suggests a heart condition cannot be managed remotely, as it requires a direct physical examination and diagnostic testing.”*

In addition, another GP emphasized the importance of clear regulations to support the effective implementation of telemedicine applications. Such regulations can serve as guidelines for physicians and telemedicine service providers to ensure that the services they deliver comply with applicable standards and laws. The GP stated:

*“… the important thing is aligning perceptions. The government or the Indonesian Medical Association must establish clear regulations regarding what can and cannot be managed through telemedicine services.”*

### Theme four: Telemedicine application utilization

Based on the interview results, most GPs were found to make good use of the features provided in telemedicine applications. They reported using all or nearly all of the available features in the telemedicine application. One GP stated:

*“I use almost all the features in the application.”*

The level of interest among GPs in exploring the features available in telemedicine applications varies. A small number of GPs reported limited interest in feature exploration and tended to use only the features necessary for consultation, as one GP explained:

“*No, I don’t really explore the features much. I only use them as needed for the consultation.”*

However, most GPs expressed a strong interest in exploring telemedicine application features. Their motivation for engaging in feature exploration includes improving the application’s quality by identifying deficiencies or potential areas for feature development; streamlining services to enhance efficiency and responsiveness to patient needs; expanding their knowledge and skills to support service delivery; and gaining a clearer understanding of the available features, including their functions, usage, and location within the application. One GP explained:

*“Of course. Because the application is like a friend to me, a partner in providing quick consultations, so exploring it is important. Knowing our tools is crucial.”*

Regarding the routine use of telemedicine applications to provide remote healthcare services, sixteen out of seventeen GPs stated that they use telemedicine applications regularly, while one GP reported otherwise. Furthermore, the frequency of telemedicine application use varied among GPs. One GP reported using the application twice a week, others used it daily or almost daily, and most GPs reported a usage frequency of approximately five times a week. As one GP shared:

*“Since I primarily work in telemedicine now, it’s about five times a week, more or less.”*

### Theme five: Performance impact of telemedicine application

Before the interviews were conducted, several performance metrics related to GPs’ use of telemedicine applications to provide remote healthcare services to patients were identified, including healthcare service quality, effectiveness, efficiency, diagnostic accuracy, decision-making quality, communication among physicians, and communication between physicians and patients. The interviews were conducted to confirm whether these performance metrics are considered important by the GPs to be achieved or improved, and to determine if any additional metrics should be included.

All participants considered healthcare service quality, effectiveness, efficiency, diagnostic accuracy, decision-making quality, and communication between physicians and patients to be important. In addition, most GPs acknowledged the importance of communication among physicians, particularly in ensuring coordinated care. However, a few GPs felt that communication among physicians was less relevant within the context of telemedicine, as one GP explained:

*“Communication among physicians seems unnecessary because we don’t communicate with each other to discuss a patient.”*

Regarding the addition of other important performance metrics, some GPs felt that the current metrics were sufficient, while others suggested the inclusion of new ones. However, the proposed metrics were related to previously identified performance metrics, such as cost containment and the speed of service delivery, which are related to healthcare service efficiency, and therapy accuracy, which relates to healthcare service effectiveness.

Most participants believed that telemedicine applications have great potential to enhance various aspects of performance in delivering remote healthcare services. Some GPs noted that telemedicine has positively impacted all aspects of performance in delivering remote healthcare services, except communication among physicians. One GP stated:

*“The quality of services, diagnostic accuracy, decision quality, physician–patient communication, as well as the effectiveness and efficiency of healthcare services have been improved. However, communication among physicians has not yet improved.”*

However, most GPs noted that telemedicine has positively impacted only several areas, such as service quality, efficiency, effectiveness, and communication between physicians and patients, as one GP said:

*“The performance that can be improved the most, in my opinion, is definitely the efficiency of health services. Besides, effectiveness, service quality, and communication between physicians and patients can also be enhanced.”*

All GPs provided various suggestions to improve the telemedicine application so it can better support their performance in delivering remote healthcare services. One of the main recommendations, as raised by most GPs, was to improve the application’s stability and performance. They emphasized the importance of reducing technical issues or disruptions that could hinder service delivery. Some GPs also suggested enhancing existing features and adding new ones that are currently unavailable, such as a referral feature integrated with healthcare facilities or laboratories, as well as other essential features that have not yet been provided. One GP expressed:

*“Yeah, hopefully there won’t be any issues with the app, and hopefully essential features, like a referral feature, can be added. My suggestion would be to reduce the occurrence of technical issues, if possible, and to improve and add more features, such as those mentioned in the previous list.”*

## Discussion

This study aimed to investigate GPs’ views on the use of telemedicine applications in providing remote healthcare services. The investigation was framed using the TTF model, which includes the aspects of task, technology, TTF, utilization, and performance impact.

### Clinical tasks performed by GPs in remote healthcare services delivery

To evaluate how effectively telemedicine applications can support GPs in providing remote healthcare services, it is essential to identify the main tasks performed by GPs in delivering such services. These tasks must be supported by the telemedicine application. The primary tasks involved in providing remote healthcare (clinical consultations) are anamnesis (taking patients’ medical history), conducting physical examinations, providing advice or education, establishing diagnoses, providing treatment plans, prescribing medications and/or medical devices, issuing referrals, conducting follow-ups, reviewing diagnostic test results, and issuing medical certificates. These findings align with the expert consultation process model developed by Serrano and Karahanna [18].

According to Serrano and Karahanna [18], an expert consultation session begins with the session initiation phase, which is establishing the reason for the consultation. This phase corresponds to the anamnesis process, during which the GP identifies the reason for the consultation. Once the problem has been stated, the information-gathering phase begins. This phase involves anamnesis, physical examination, and reviewing diagnostic test results, allowing the GP to collect information about the patient’s health condition. Next, in the analysis and diagnosis phase, the GP analyzes the exchanged information and establishes a diagnosis. In the explanation and planning phase, the GP communicates the solution to the patient’s health issues and recommends appropriate actions, which may include giving advice or education, outlining a treatment plan, prescribing medications and/or medical devices, or issuing a referral. The consultation session may then conclude with the issuance of a medical certificate or the scheduling of a follow-up, if needed, which corresponds to the close session phase.

Based on the identified tasks, several characteristics of GPs’ tasks in delivering remote healthcare services through telemedicine applications can be discerned. One such characteristic is time criticality, as GPs often require rapid access to data and must make immediate decisions regarding patient diagnosis and treatment [40,52,53]. A second characteristic is interdependence, as GPs may need to collaborate with other healthcare professionals and handle issues that often involve more than one healthcare function, thus requiring the telemedicine application to support effective communication and data integration [15,40,53]. Another characteristic is data dependency, since GPs need access to patient data to support timely and accurate medical decision-making [53].

Task characteristics are one of the key predictors of TTF [54]. Prior studies have empirically examined the influence of task characteristics on TTF and found that task characteristics significantly influence TTF (e.g., [39,40,54,55]). The findings of this study extend the TTF literature by illustrating how task characteristics manifest in the context of telemedicine-based remote healthcare delivery from the physicians’ perspective. Specifically, the identified clinical tasks are characterized by time criticality, interdependence, and data dependency, which place distinct demands on telemedicine applications. These findings provide a conceptual foundation for subsequent empirical examinations of the relationships between task characteristics and TTF in telemedicine contexts.

### Telemedicine application requirements

Each task performed by physicians in providing remote healthcare services needs to be supported by the telemedicine application they use. Each application has requirements that define what it must do and the properties or qualities it must possess [56].

One essential requirement is data and services integration, which requires telemedicine applications to have integrated data and the ability to seamlessly connect with other systems to ensure data interoperability. Khodaveisi et al. [57] note that telemedicine applications must have the ability to connect with Health Information Systems (HIS), Electronic Health Records (EHR), Personal Health Records (PHR), as well as laboratory and radiology information systems. Such integration allows telemedicine applications to access patient medical records, clinical data, and supporting information needed for effective consultation and diagnosis [58]. Haleem et al. [58] explain that consolidating patient records into a single system enables telemedicine applications to facilitate streamlined coordination among physicians, pharmacies, and other healthcare providers, ultimately improving the speed and efficiency of healthcare delivery. Integration also enables medical devices and wearables, such as pulsometers and blood pressure monitors, to transmit vital signs and biometric data directly to clinicians, supporting real-time monitoring and timely treatment adjustments [58].

Clinical decision support is another key requirement, as it provides physicians with recommendations for diagnosis, treatment, and patient care, and helps to prevent prescription errors by considering drug interactions and patient-specific conditions. Telemedicine solutions play a significant role in supporting clinical decision-making and their effectiveness is further enhanced when integrated with artificial intelligence (AI), enabling more thorough evaluations and detection of abnormalities [58]. AI can help physicians and other medical professionals make clinical decisions more quickly and accurately [59].

Clinical communication is equally vital, enabling effective information exchange between physicians, patients, and other healthcare professionals through voice, video, or text messaging [60,61]. Through telemedicine, physicians can remotely collect and exchange information with their patients [58]. Patients can communicate symptoms, ask questions, and upload medical test reports or diagnostic images as needed for evaluation and treatment [57,62]. Telemedicine also facilitates physician-to-physician communication, allowing the exchange of patient history, medical reports, and diagnostic images with specialists in different locations [58]. Haleem et al. [58] emphasize that virtual consultations support collaborative care, allowing primary care physicians to seek expert advice when facing diagnostic or treatment concerns, with specialists providing feedback electronically or through virtual meetings. This process reduces unnecessary in-person referrals, shortens wait times for specialist input, and minimizes patient travel [58].

Clinical task support ensures that the application can support the range of clinical tasks performed by physicians, such as generating referral letters and structured prescriptions. The referral letter is an important document facilitating the transfer of care from a GP to secondary care [63], yet issues with appropriateness, clarity, and completeness remain common [64]. Several studies have demonstrated that the use of referral templates enhances the quality, clarity, and completeness of primary care referrals [63,64]. Similarly, incomplete or illegible prescriptions can lead to serious medication errors, posing significant risks [65]. Raza et al. [65] explain that the implementation of structured prescription forms enhances the completeness and legibility of written prescriptions, thereby improving their quality.

Scheduling and notification features support the organization of consultations and follow-up appointments, while also providing reminders or alerts to both physicians and patients. Haleem et al. [58] emphasize that scheduling and rescheduling are standard features of modern telemedicine applications, enabling physicians to efficiently manage appointments. Haleem et al. [58] also notes that appointment scheduling functions keep physicians informed of their appointment status. In addition, standard notification features available in the application ensure that relevant users receive timely alerts [66]. These alerts may include reminders for patients to arrange a doctor’s appointment [60].

System reliability is essential to ensure that telemedicine applications remain active and available when needed and free from technical disruptions that could hinder service delivery. Rehman and Khan [62] emphasize that a telemedicine application must be reliable because the service it provides can be critical in saving lives. If the service is unavailable when most needed, its purpose is undermined [62]. Rehman and Khan [62] further note that to maintain reliability, systems must incorporate mechanisms such as fault tolerance to ensure continuous operation. Additionally, achieving reliability requires prompt response times when encountering errors, timely repairs and updates, and efforts to minimize the frequency and severity of system errors [57].

Ease of use and support are crucial to ensure that telemedicine applications can be easily learned and operated, and users receive sufficient assistance when they encounter difficulties in navigating or using the system. A user-friendly system should be simple and intuitive for users [67], supported by accessible training and learning processes to facilitate its adoption and use [57]. Effective customer support is also critical [67], including the availability of support teams that can be contacted to resolve technical issues promptly [60].

Privacy and security ensure the protection of patient data in telemedicine applications by implementing mechanisms that restrict unauthorized access and prevent data breaches. Privacy and security are crucial requirements for telemedicine systems, given the highly sensitive nature of patient health data [62,68]. Vulnerabilities regarding privacy and security can lead to breaches in the confidentiality of patients’ data, resulting in stress, dissatisfaction, or even delays in seeking effective treatment due to perceived privacy risks [69]. A security requirement can be defined as a control, safeguard, or countermeasure that prevents or eliminates vulnerabilities that could compromise the confidentiality, integrity, or availability of data [62]. In practice, data protection is often achieved through cloud-based medical record-keeping, which securely stores patient information and captured video consultations [58].

Finally, data quality and accessibility ensure that the information stored within the application is accurate, up-to-date, consistent, and readily available to authorized users. A high-quality system stores data at an appropriate level of detail, integrates data from various sources without inconsistencies, and presents information in a clear, readable format [70]. Users should be able to easily locate, access, and retrieve the required data whenever needed [62,70].

In this study, the identified telemedicine application requirements represent the technology aspect of the TTF model. These requirements provide an overview of the essential aspects that must be present in such applications to support GPs in delivering remote healthcare services and can serve as a foundation for developing an instrument to evaluate the TTF of telemedicine applications.

From a practical perspective, these findings provide actionable guidance for telemedicine developers and policymakers. Developers can use the identified requirements as design priorities to ensure that telemedicine applications adequately support physicians’ clinical workflows. Policymakers and regulators may also use these findings to establish minimum functional and quality standards for telemedicine platforms, ensuring that essential features are consistently available across providers.

### Task-technology fit of telemedicine application

The findings indicated that telemedicine applications, with their available features, can generally support the tasks involved in providing remote healthcare services. However, there are still shortcomings in the applications that need to be addressed. This highlights the importance of conducting a TTF evaluation to identify areas that require improvement, so that telemedicine applications can fully support GPs in delivering remote healthcare services.

TTF can be assessed using a set of questionnaire items answered by individuals performing tasks with the aid of a technology [71]. A questionnaire-based TTF evaluation instrument can help physicians assess how well a telemedicine application supports their tasks while also providing valuable feedback to developers on areas that need improvement. Currently, there is no questionnaire specifically designed to measure the extent to which technology supports physicians’ tasks in the context of telemedicine [72]. Junglas et al. [16] emphasize that there is no universal concept of fit applicable to all situations and therefore any TTF evaluation instrument must be tailored to its specific context of use [71]. Additionally, El-Gayar et al. [73] note that while existing TTF instruments have demonstrated promising results in healthcare settings, further research is needed to adapt these instruments to meet user needs through the development of context-relevant task models. Therefore, further research is necessary to develop a TTF evaluation instrument that considers the specific tasks carried out by physicians in delivering remote healthcare services [72].

Nevertheless, it is important to acknowledge that telemedicine applications will inevitably have limitations, so not all patient cases can be managed through telemedicine services. Thus, in addition to TTF evaluation, clear regulatory guidelines established by policymakers and regulators are also needed to define the scope of services and the boundaries of care that can be appropriately delivered through telemedicine.

### Telemedicine application utilization

TTF evaluation is important because TTF is considered a key factor that influences both the utilization of information technology and its performance impact [39]. In general, utilization is defined as the extent to which technology is integrated into the work processes or tasks performed by users [40]. According to O’Connor et al. [40], utilization is not limited to general technology use but includes several specific aspects such as routine use, feature use, and value-adding use. Routine use refers to the extent to which physicians automatically use the application as part of their daily practice. Feature use describes the extent to which physicians utilize the features or functions within the application to complete certain tasks. Meanwhile, value-adding use involves additional usage that enhances the outcomes or the impact of the technology on healthcare services.

Regarding routine use, the frequent and regular use of telemedicine applications by most GPs indicates that these systems have been integrated into their medical practice. Almost all available features are used by GPs, likely because the telemedicine applications currently in use only offer standard features that are essential for delivering remote healthcare services. The level of feature exploration among GPs varied, but the majority expressed an interest in exploring the application further, for example, to better understand system functions, improve service efficiency, and identify potential areas for improvement. This exploration reflects the aspect of value-adding use, in which GPs not only rely on the basic features but also actively seek to optimize the application’s use to support their clinical practice.

The aspects of feature use and value-adding use in the study by O’Connor et al. [40] are related to the concept of innovative use discussed in the study by Li et al. [74]. These aspects pertain to the post-adoption phase of a technology [40,74]. Unlike the adoption phase, which focuses on initial acceptance and the user’s decision to begin using the technology, the post-adoption phase centers on the continued use of the technology. Post-adoption behavior refers to an individual’s decision to continue using a technology or information system beyond initial use and to further exploit and expand the functionalities built into that technology or system [75]. Continued use of information systems is critical for the success of system implementation, as the benefits of an information system investment can only be realized if the system is used on an ongoing basis [76,77].

### Performance impact of telemedicine application

Many previous studies on technology adoption or acceptance focused solely on variables related to adoption or post-adoption behavior as dependent variables when examining the factors that influence the acceptance or adoption of a particular technology [10,19,21–24]. However, Isaac et al. [78] argue that it is also important to emphasize the outcomes of using a technology by considering performance impact in evaluating and measuring its success. Within the TTF framework, performance impact represents the outcome that arises when technology effectively supports task requirements, making it a critical indicator of telemedicine success from the physicians’ perspective.

The expected performance impact of using a particular technology may vary depending on its type, intended use, and the context in which it is applied. For instance, Isaac et al. [79] define the performance impact of internet use as the extent to which it influences decision quality, job processes, and knowledge acquisition. In the present study, the performance impacts expected by GPs from using a telemedicine application to provide remote healthcare services include improvements in healthcare service efficiency, effectiveness, and quality, as well as diagnostic accuracy, decision-making quality, communication between physicians and patients, and communication among physicians.

Telemedicine enables broader and more equitable access to healthcare services, especially for patients in rural or remote areas [80]. It also offers better healthcare options and contributes to cost savings for both physicians and patients by optimizing clinical procedures and reducing travel expenses to healthcare facilities [58]. Telemedicine has proven to be cost-effective and requires only minimal equipment for accessibility, typically only a smartphone [81]. When implemented in the appropriate context, telemedicine does not reduce the effectiveness of clinical care compared to conventional healthcare services [82]. Alfarwan et al. [7] even state that telemedicine is as effective as conventional care in improving patient health outcomes. In light of these advantages, GPs expect that the use of telemedicine applications for providing remote healthcare services will maintain or even enhance the efficiency and effectiveness of healthcare delivery.

Moreover, telemedicine allows patients to receive medical attention at a time and in a manner that is convenient for both themselves and their physicians, while also ensuring safety [58]. Kruse et al. [80] further emphasize that telemedicine enables higher-quality care, improves patient safety, and reduces the risk of medical errors. With the advancement of internet technology, digitalization has become increasingly integrated into telemedicine [83] and this is expected to help improve diagnostic accuracy and the quality of clinical decision-making. Consequently, GPs anticipate that the use of telemedicine applications can maintain or even improve the quality of healthcare services, diagnostic accuracy, and clinical decision quality.

Nevertheless, telemedicine also presents challenges, including the potential reduction in the sense of connection between physicians and patients, as well as difficulties in sharing information and engaging in shared decision-making [84]. Therefore, communication features within the telemedicine applications are expected to facilitate more effective interactions with patients. Furthermore, physicians can utilize telemedicine to share expertise and build support networks [58]. However, some GPs feel that communication among physicians is not particularly relevant in the context of telemedicine. They argue that they rarely communicate with other physicians when managing a patient, as telemedicine consultations typically consist of individual interactions between the physician and the patient. Nonetheless, physician-to-physician communication remains important in telemedicine, especially in cases involving patient referrals. Although it often does not involve direct communication between physicians, referral processes still require one physician to convey clinical information to another. This can occur, for example, when GPs refer patients to medical specialists, using referral letters [85] or electronic health records [86]. This form of communication is crucial for maintaining continuity of care.

The findings indicate that, although telemedicine applications have positively impacted various performance aspects, such as healthcare service efficiency, effectiveness, quality, and communication between physicians and patients, there is still room for improvement, particularly in enhancing decision-making quality, diagnostic accuracy, and communication among physicians. Several recommendations were identified to optimize telemedicine applications in supporting physicians’ performance in remote healthcare delivery. One key area requiring improvement is system stability and reliability, as some GPs frequently experience technical issues that disrupt the service continuity. Additionally, enhancing existing features and developing new ones to better support clinical tasks were emphasized. By addressing technical issues and developing application features tailored to physicians’ needs, telemedicine applications have great potential to further enhance physician performance in delivering remote healthcare services.

### Limitations and strengths

This study had several key strengths. The use of a qualitative approach allowed for an in-depth exploration of GPs’ perspectives and experiences with telemedicine applications, offering richer insights into how telemedicine applications support physicians’ tasks, enhance their performance, and address unmet needs compared to previous quantitative studies. Additionally, this was the first qualitative study in Indonesia, a developing country [28], to comprehensively apply the TTF model to evaluate task, technology, TTF, utilization, and performance impact aspects of telemedicine applications. This study also identified various tasks and system requirements necessary for telemedicine applications to effectively support GPs’ clinical work, which can serve as a foundation for developers to enhance their features and services. The interviews were conducted with GPs who actively use telemedicine applications in their daily practice, ensuring that the data collected reflects real-world experiences rather than theoretical assumptions. Furthermore, the rigorous analytical methodology—employing both deductive and inductive content analysis, supported by the use of the Atlas.ti software—strengthens the transparency and validity of the study’s findings.

Despite these strengths, several methodological limitations should be acknowledged. First, all interviews were conducted remotely, which may have limited the capture of non-verbal cues and contextual information that could have enriched data interpretation. To mitigate this limitation, interviews were conducted synchronously using video conferencing, allowing for real-time interaction, and participants were encouraged to provide detailed explanations and concrete examples. Second, interview transcripts were not returned to participants for review, and formal participant feedback on the findings was not obtained. The absence of transcript feedback may have limited opportunities for participants to clarify or elaborate on their statements. Despite this, all interviews were audio-recorded and carefully transcribed to ensure data accuracy. Third, the findings rely on participants’ self-reported experiences, which may be subject to recall bias. This risk may be partially mitigated by the fact that most participating GPs reported using telemedicine applications regularly, often daily or several times per week, suggesting that their accounts were based on recent and routine practice rather than distant recollections. Fourth, the data analysis was conducted by a single researcher (single coder), while involving multiple coders could have further strengthened the rigor of the study’s findings. Nonetheless, the analysis was conducted carefully following a systematic content analysis procedure with clearly defined steps to enhance consistency and transparency.

In addition, only GPs were interviewed, thereby excluding the perspectives of specialists, who may have different experiences and needs when using telemedicine applications. Furthermore, the study was conducted in collaboration with Good Doctor, which provided access to the participating GPs. As a result, the insights primarily reflect the views of GPs affiliated with a single telemedicine provider. Nevertheless, as several participants had experience with multiple telemedicine applications, the insights derived from this study extend beyond Good Doctor and are applicable to telemedicine applications in general.

## Conclusions

This study contributes to the telemedicine and TTF literature by employing the TTF model, which encompasses task, technology, TTF, utilization, and performance impact aspects, to qualitatively examine the use of telemedicine applications by GPs in delivering remote healthcare services. The use of a qualitative approach allowed for an in-depth exploration of GPs’ perspectives and experiences with telemedicine applications, offering richer insights compared to previous quantitative studies. By capturing GPs’ experiences, this study extends prior research through the identification of context-specific clinical tasks, technology requirements, and performance expectations in telemedicine practice.

The findings highlight that while telemedicine applications generally support physicians’ clinical tasks and contribute to improved efficiency, effectiveness, service quality, and physician–patient communication, important gaps remain. These gaps relate primarily to system integration, limitations in conducting comprehensive physical examinations, and challenges in supporting diagnostic accuracy, clinical decision-making, and physician-to-physician communication.

From a practical perspective, this study provides actionable guidance for telemedicine developers and policymakers. The identified application requirements can inform design priorities aimed at better supporting physicians’ clinical workflows, while policymakers and regulators can use these findings to establish regulatory guidelines regarding the scope of services and standards for safe and standardized telemedicine practice.

Despite its contributions, this study has several methodological limitations, including the use of remote interviews, the absence of transcript feedback, potential recall bias, the use of a single coder, a limited participant scope, and recruitment through a single telemedicine provider. This study opens several directions for future research. Building on the identified tasks and system requirements, future studies could focus on developing and validating an evaluation instrument for assessing the TTF of telemedicine applications from physicians’ perspectives. In addition, future studies could broaden the scope of this study by including specialists, patients, or other stakeholders to capture a more holistic perspective.

## Supporting information

S1 File Interview guide. (DOCX)

S1 Checklist Consolidated criteria for reporting qualitative research (COREQ). (PDF)

S1 Table The description of each identified telemedicine application requirement. (DOCX)
