# Supplementary material for: Using the task-technology fit model to examine the use of telemedicine applications by general practitioners in Indonesia: A qualitative study
Source: PLoS One. 2026 Jun 8;21(6):e0351130. doi: 10.1371/journal.pone.0351130 (PMC13245790; doi:10.1371/journal.pone.0351130)
Supplement: S1 Table — (DOCX) [file pone.0351130.s003.docx]

**S1 Table. The description of each identified telemedicine application requirement.**

| Task | No. | Requirement | Description |
| --- | --- | --- | --- |
| Anamnesis | 1 | Real-time communication | Real-time communication via audio, video, and/or chat is required to support communication between the physician and the patient |
|  | 2 | Upload images | Uploading images or documents is required to allow patients to upload lab results, X-rays, or other medical documents to support anamnesis |
|  | 3 | Upload documents |  |
|  | 4 | Access to patient data | Access to patient data is required so that physicians can easily access the patient's medical records to support anamnesis |
|  | 5 | Integration with other platforms | Integration with other platforms, such as hospital information systems or laboratories, is required to enable physicians to easily access relevant information to support anamnesis |
|  | 6 | Patient medical data recording | Medical data recording is required to document patient information during the consultation |
|  | 7 | Question template^a^ | Question templates can help physicians collect information from patients in a structured, systematic, and efficient manner |
| Conducting physical examinations | 1 | Record and store | The record and store function is required to record examination sessions and save them in accordance with regulatory requirements |
|  | 2 | Integration with medical devices | Integration with medical devices, such as digital blood pressure monitors, digital thermometers, or other vital sign monitoring tools capable of transmitting real-time data, is required to support physical examinations. |
|  | 3 | Real-time communication | Real-time communication via audio and/or video is required to allow physicians to examine patients and provide direct feedback or instructions during the examination |
|  | 4 | Upload short audio^a^ | Uploading short audio is required to allow patients to send sound recordings—such as cough sounds, breathing sounds, or others—to support physical examinations by physicians |
|  | 5 | Upload images^a^ | Uploading images is required to allow patients to send photos of specific body areas—such as skin, eyes, or others—to support physical examinations by physicians |
|  | 6 | Upload short video^a^ | Uploading short videos is required to allow patients to show specific conditions, expressions, or movements to support physical examinations by physicians |
| Providing advice or education | 1 | Integration of examination data | Integration of examination data is required so that the results of diagnostic tests (such as laboratory tests, radiology, etc.) and/or physical examinations can be integrated and presented to the physician |
|  | 2 | Referral function | If the patient needs to be referred to another healthcare facility, the referral function is required to enable the physician to provide a digital referral through the application |
|  | 3 | Access to healthcare facility data | Access to healthcare facility data allows physicians to view a list of nearby or relevant healthcare facilities, enabling them to provide recommendations to patients on where to go for further examination or treatment |
|  | 4 | Advice/ education documentation | This documentation is required to record the advice or education provided, for future reference |
|  | 5 | Recommendation function | This function can assist physicians in providing advice or education to patients based on examination results and patient data |
|  | 6 | Real-time communication | Real-time communication via audio, video, and/or chat can help physicians provide recommendations or advice to patients easily |
| Establishing diagnoses | 1 | Recommendation function | This function can assist physicians by providing diagnostic suggestions or recommendations based on information provided by the patient and examination findings |
|  | 2 | Real-time communication | Real-time communication via audio, video, and/or chat can facilitate dialogue between the physician and the patient, allowing the physician to ask follow-up questions or request clarifications that can support the diagnostic process and/or deliver the diagnosis |
|  | 3 | Collaboration function | In complex diagnostic cases, the collaboration function allows physicians to consult with specialists or colleagues to obtain a second opinion |
|  | 4 | Medical data access and integration | Access and integration of medical data are required so that the results of anamnesis, physical examinations, and diagnostic tests can be integrated and accessed within a single platform, making it easier for physicians to establish a diagnosis |
|  | 5 | Access to medical database | Access to the medical database allows physicians to retrieve information about diseases or other medical conditions, such as their signs and symptoms, thereby supporting the diagnostic process |
|  | 6 | Reporting feature | After a diagnosis is established, the reporting feature enables physicians to prepare and provide medical reports to the patient |
|  | 7 | Complete ICD-10 list^a^ | ICD-10 is a medical classification list issued by the WHO, used to categorize and code various types of diseases, disorders, and symptoms. It is required to maintain consistency and assist physicians in documenting and reporting diagnoses |
| Providing treatment plans | 1 | Prescription recording | Prescription recording enables physicians to provide prescriptions to patients |
|  | 2 | Patient monitoring | Patient monitoring enables physicians to track the patient's progress after therapy or treatment has been administered |
|  | 3 | Referral function | The referral function enables physicians to digitally refer patients to specialists or other healthcare facilities |
|  | 4 | Treatment notes | Treatment notes enable physicians to document the patient's treatment plan, the patient's response, and any necessary adjustments |
|  | 5 | Recommendation function | The recommendation function can assist physicians by providing treatment options based on patient data and diagnostic information |
|  | 6 | Real-time communication | Real-time communication via audio, video, and/or chat enables physicians to communicate with patients about the treatment plan, side effects, response to treatment, or other related matters |
|  | 7 | Collaboration function | The collaboration function allows physicians to consult or collaborate with specialists or colleagues to discuss the patient's treatment plan |
|  | 8 | Feedback and evaluation | Feedback and evaluation allow patients to provide assessments of the services delivered by the physician or share feedback on their response to the treatment. |
|  | 9 | Informed consent^a^ | Informed consent is the process of delivering information by the physician to the patient about the benefits, risks, and alternatives of a medical recommendation. This requirement enables the patient to provide written approval or refusal to the recommendation. |
| Prescribing medications and/or medical devices | 1 | Prescription template | The prescription template enables physicians to issue prescriptions quickly, accurately, and consistently by providing a standardized and editable format |
|  | 2 | Open loop prescribing | Electronic prescribing of medications and/or medical devices can be conducted in an open-loop manner, where the electronic prescription is given to the patient to be submitted to the pharmacy facility |
|  | 3 | Electronic prescription identification code | Open-loop electronic prescribing requires an electronic prescription identification code that can be verified for authenticity and validity by the pharmacy facility |
|  | 4 | One-time use | The electronic prescription is intended for one-time use only and cannot be repeated (non-repetitive) |
|  | 5 | Access to medications and/or medical devices data | The application should have an integrated database of medications and medical devices |
|  | 6 | Electronic prescription documentation | The electronic prescription must be storable as part of the patient's medical record documentation |
|  | 7 | Closed loop prescribing | The electronic prescription is transmitted directly from the physician to the pharmacy through the application |
|  | 8 | Integration with pharmacy facilities | Closed-loop electronic prescribing is carried out through the application from the physician to the pharmacy facility, thus requiring integration with pharmacy facilities |
|  | 9 | Recommendation function | The recommendation function can provide suggestions or alerts to physicians when writing prescriptions, for example, if the prescribed medication has potential interactions with other drugs the patient is currently taking |
|  | 10 | Automated medication restriction for drugs excluded by Ministry of Health regulations | The system automatically prevents the prescription of medications excluded by Ministry of Health regulations, such as narcotics and psychotropic drugs, injectable medications (except insulin for self-use), and contraceptive implants |
|  | 11 | Automated medication restriction to prevent the prescription of drugs that are contraindicated for specific patient conditions^a^ | The system automatically prevents the prescription of medications that are not suitable for patients with special conditions—such as pregnancy, breastfeeding, kidney dysfunction, allergies, or other medical conditions—ensuring that only safe and appropriate medications can be prescribed |
| Providing referrals | 1 | Referral letter template | Referral letter template provides a standardized and customizable format that enables physicians to issue referral letters in accordance with medical standards |
|  | 2 | Access to patient data | Access to patient data enables physicians to easily retrieve relevant patient information needed to complete and issue accurate referral letters |
|  | 3 | Integration with healthcare facilities | Integration with healthcare facilities allows physicians to seamlessly select and refer patients to appropriate healthcare facilities directly through the application |
|  | 4 | Referral history | Referral history enables access and storage of previously issued referral letters for reference and documentation purposes |
|  | 5 | Collaboration function | The collaboration function allows the referring physician to share information about the patient and their health condition with the receiving physician or healthcare facility; likewise, the receiving party can provide updates or examination results back to the referring physician |
| Conducting follow-ups | 1 | Follow-up note^a^ | The follow-up note allows physicians to record the follow-up date and explain the follow-up needs to the patient, and it can also be used by the patient to report their response to treatment and any changes in condition after the consultation |
|  | 2 | Real-time communication^a^ | Real-time communication via chat, audio, and/or video calls enables physicians and patients to interact directly, discuss the patient's condition, evaluate treatment progress, and provide medical advice |
|  | 3 | Upload images^a^ | Image upload allows patients to send images or photos to support the follow-up task |
|  | 4 | Reminder^a^ | Reminder is a notification sent to the patient to remind them of their follow-up schedule |
|  | 5 | Follow-up scheduling^a^ | Follow-up scheduling allows physicians to schedule follow-up appointments with patients |
| Reviewing diagnostic test results | 1 | Referral function^a^ | If the patient needs to be referred to another healthcare facility, the referral function is required to enable the physician to issue a digital referral through the application |
|  | 2 | Diagnostic test history^a^ | Diagnostic test history allows the results of diagnostic tests to be stored and easily accessed by both physicians and patients through the telemedicine application |
|  | 3 | Upload documents^a^ | Document upload allows patients to upload diagnostic tests result documents |
|  | 4 | Upload images^a^ | Image upload allows patients to upload images of diagnostic tests results |
| General task | 1 | System reliability | The telemedicine application must be reliable, consistently active and available when needed, without frequent issues or system failures, and free from unexpected downtimes that could disrupt the delivery of remote healthcare services |
|  | 2 | Ease of use | The telemedicine application is easy to learn and use |
|  | 3 | Assistance | The telemedicine application provides users with easy access to support for issues related to the computer system and data |
|  | 4 | Authorization | The telemedicine application provides verification and validation mechanisms to ensure that only authorized individuals can access the data |
|  | 5 | Privacy and security | The telemedicine application has strong security features to protect data from unauthorized access and data breaches |
|  | 6 | Right level of detail | The telemedicine application stores data at an appropriate level of detail |
|  | 7 | Currency | The telemedicine application provides sufficiently up-to-date data |
|  | 8 | Accuracy | The telemedicine application provides accurate data |
|  | 9 | Compatibility | Data from various sources can be integrated or compared without inconsistencies |
|  | 10 | Accessibility | The data needed in the telemedicine application is easily accessible |
|  | 11 | Locatability | Users can easily identify what data is available and where it can be found |
|  | 12 | Presentation | Data in the telemedicine application is presented in a format that is easy to read and understand |
|  | 13 | Notification | Notifications allow physicians to receive immediate alerts about consultation schedules, schedule changes, or consultation requests. They can also be used for security purposes, such as notifying login attempts from unfamiliar devices or locations |
|  | 14 | Scheduling | Scheduling allows physicians to manage consultation appointments with patients |

^a^The requirement was added by the participant.
